# Comparative study of the methane production based on the chemical compositions of *Mangifera Indica* and *Manihot Utilissima* leaves

**DOI:** 10.1186/s40064-015-0832-y

**Published:** 2015-02-11

**Authors:** Philippe Mambanzulua Ngoma, Serge Hiligsmann, Eric Sumbu Zola, Marc Culot, Thierry Fievez, Philippe Thonart

**Affiliations:** Walloon Center of Industrial Biology (CWBI), Gembloux Agro-Bio Tech, University of Liège, 2 Passage des Déportés, 5030 Gembloux, Belgium; Faculty of Pharmaceutical Sciences, University of Kinshasa, P. O. Box 212, Kinshasa XI, Democratic Republic of Congo; Faculty of Agricultural Sciences, University of Kinshasa, P. O. Box 117, Kinshasa XI, Democratic Republic of Congo; Laboratory of Microbial Ecology and Water Purification, Gembloux Agro-Bio Tech, University of Liège, B52, 27 Maréchal Juin, B-5030 Gembloux, Belgium

**Keywords:** Anaerobic digestion, Biogas, Methane, Leaves, *Mangifera Indica*, *Manihot Utilissima*

## Abstract

Leaves of *Mangifera Indica* (MI, mango leaves) and *Manihot Utilissima* (MU, cassava leaves) are available in tropical regions and are the most accessible vegetal wastes of Kinshasa, capital of Democratic Republic of Congo. These wastes are not suitably managed and are not rationally valorized. They are abandoned in full air, on the soil and in the rivers. They thus pollute environment. By contrast, they can be recuperated and treated in order to produce methane (energy source), organic fertilizer and clean up the environment simultaneously. The main objective of this study was to investigate methane production from MI and MU leaves by BMP tests at 30°C. The yields achieved from the anaerobic digestion of up to 61.3 g raw matter in 1 l medium were 0.001 l/g and 0.100 l CH_4_/g volatile solids of MI and MU leaves, respectively. The yield of MU leaves was in the range mentioned in the literature for other leaves because of a poor presence of bioactive substrates, and low C/N ratio. This methane yield corresponded to 7% of calorific power of wood. By contrast, the methane yield from MI leaves was almost nil suggesting some metabolism inhibition because of their rich composition in carbon and bioactive substrates. Whereas classical acidogenesis and acetogenesis were recorded.

Therefore, methane production from the sole *MI* leaves seems unfavorable by comparison to *MU* leaves at the ambient temperature in tropical regions. Their solid and liquid residues obtained after anaerobic digestion would be efficient fertilizers. However, the methane productivity of both leaves could be improved by anaerobic co-digestion.

## Introduction

Kinshasa (capital of Democratic Republic of Congo) has a tropical climate and the majority of wastes are of vegetal origin (Nzuzi 1999; Biey 2013, General Director of the “Régie d’Assainissement des Travaux Publics de Kinshasa”, Kinshasa/Lingwala, Democratic Republic of Congo, personal communication). These wastes are not suitably managed and are not rationally valued. They are abandoned in public trash cans, thrown in rivers or gutters, becoming thus the foyers of proliferation of microbes and the vectors of diseases. Whereas this biomass can be recovered and treated by anaerobic biodegradation for producing methane. The anaerobic digestion of vegetal wastes at ambient temperature could be a favorable treatment mode for the cleaning up of the Kinshasa environment. It would reduce energy consumption for heating the reactor and the costs of facilities (Mambanzulua et al. 1999; Kamdem et al. 2011). This biological process results in methane production as source of energy and residues or digestates as fertilizing matters.

According to many authors, solid wastes of vegetal origin represent a high potential energy resource if they can be properly and biologically converted to methane (Gunaseelan 2004; Barakat et al. 2012; Chandra et al. 2012; Kamdem et al. 2013). They are renewable and therefore their net CO_2_ contribution to the atmosphere is nil.

In this work, we are interested in the methane production from *Mangifera Indica* (MI) and *Manihot Utilissima* (MU) leaves since large amounts of this organic matter are available in Kinshasa and other African regions and moreover, up to date, they are about not valorized. Anaerobic digestion is less prevalent in all these regions according to Vögeli et al. (2014). Consequently, very few reports of the biomethanation of these leaves have been published. High-solids digestion experiments with mango leaves and cattle manure in 1.2 m^3^ batch digesters were achieved and the biogas yield of the blend was higher than cattle manure alone (Shyam and Sharma 1994; Gunaseelan 1997). Moreover, to the best of our knowledge, the biomethanation potentials of MU and MI leaves are not still known and there has been no comparative study of the methane production based on the chemical composition of these leaves. However, before all anaerobic digestion in large scale, the biomethanation potential of feedstock must be known in order to determine the load rate, the retention time and the yield. In addition, biogas yield and its composition are greatly affected by the C/N ratio, the contents in mineral elements and in secondary metabolites in leaves (Mital 1996; Macheboeuf et al. 2011; Patra and Saxena 2010; Kamra et al. 2008).

Therefore, in this paper, a comparative biomethanation and biochemical analysis study was performed to better understand the anaerobic digestion process from MI and MU leaves and to assess their methane production potentials. The biochemical methane potential (BMP) assays were used in our experiments (Owen et al. 1979; Gunaseelan 2004) and they were carried out with monitoring of biogases volumes and their compositions, and volatile fatty acids production. After BMP assays, the fertilizing values of biomethanation residues were determined by considering the C/N ratio.

## Materials and methods

### Source and conservation of leaves

The leaves of MU and MI were collected in Kinshasa, Democratic Republic of Congo at the Rond-point Ngaba market and at the Quarter 9 in Ndjili Commune, respectively. These leaves were identified by the Herbarium of Kinshasa at the Department of Biology, Kinshasa University in Democratic Republic of Congo. These samples were washed, dried at the ambient temperature, ground and stored at 4°C in plastic bags for analyses and tests.

### Physico-chemical analyses of leaves

#### General characterization

The contents of dry matter, ash and organic matter were determined according to the standard methods (APHA 1992). The dry weight was determined by drying the sample at 105°C until a constant weight. Then, the ash content was determined by heating the dry sample at 600°C until a constant weight. The content of organic matter or, volatile solid, was calculated by the difference between the dry weight and the ash weight. The contents in Ca, Mg, K, Cu and Zn were determined by an atomic absorption spectrophotometer Perkin Elmer AAnalyst 200 (Mbonigaba 2007; Mulaji 2011). The total P, Fe and Mn were determined by an UV-visible molecular absorption spectrophotometer Unicam Helios Alpha (NF T 90–024 1963; APHA 1992; Rodier 1966). The total organic carbon (TOC) was determined by oxidization with potassium dichromate and concentrated sulfuric acid (Mulaji 2011). The total Kjeldahl N was determined by Kjeldahl method (Mze 2008).

#### Bioactive substances and total and specific polyphenols in leaves

##### Bioactive substances

Except saponins, all the active chemical groups in the aqueous extracts of leaves were identified by qualitative colorimetry after the following reactions (Lusakibanza 2012; Wagner and Bladt 1966; Angenot 1973; Vanhaelens 1994). The aqueous extracts were obtained after steeping under magnetic agitation of 25 g of leaves gunpowder in 400 ml of distilled water during 30 minutes and filtration on membrane.

The saponins were determined by vigorously agitating 5 ml of aqueous extracts in a test tube and formation of persistent foam of at least 1 cm height during 15 minutes. This test is semi quantitative method (Multon 1991).

The alkaloids were tested by adding some drops of Dragendorff reagent to 3 ml of aqueous extracts, slightly acidified. Positive test was indicated by apparition of an orange-red precipitate.

The flavonoids were identified by adding some drops of reagents of Shinoda, Mg powder and some drops of iso-amylic alcohol to 3 ml of aqueous extracts. The obtained mixture was agitated and let to rest. The presence of flavonoids was indicated by the purplish red color to the red cherry.

The anthraquinones (bound quinones) were identified by energetically mixing the reagent of Borntrager (NaOH 10%) with 3 ml of aqueous extract and apparition of a red color.

The tannins were determined by adding 1 ml of FeCl_3_ 2% (Burton reagent) to 2 ml of aqueous extract and a greenish red coloration with or without precipitate, indicates the presence of tannins. The catechic tannins were tested by adding 2 ml of Stiasny reagent to 2 ml aqueous extract. The obtained mixture was heated for 30 minutes in water bath at 90°C. A brown precipitate indicated the presence of catechic tannins. The mixture was furthermore filtered and saturated with crystals of CH_3_COONa. The presence of gallic tannins was indicated by a blackish color after addition of 1 ml of FeCl_3_ 2%.

The anthocyanins were tested by adding 2 ml of HCl 20% to 3 ml aqueous extracts. Anthocyanin chlorides crystallize with a dark pink to purplish red coloration with increasing temperature.

The leuco-anthocyanins were determined by adding some drops of Shinoda reagent to 3 ml aqueous extracts, then a small quantity of iso-amylic alcohol to develop a purplish coloration in presence of the compound.

#### Total and specific polyphenols

The concentration of water soluble total polyphenols was determined according to a procedure derived from Singleton and Rossi (1965): in a 25 ml vial, 0.5 ml of aqueous extracts of leaves 1% reacted for 3 min with 0.5 ml of Folin-Ciocalteu reagent (VWR Prolabo). After addition of 4 ml sodium carbonate solution (1 M) the mixture was brought to volume with demineralized water and homogenized. The absorbance was read at 765 nm after incubation at room temperature for 2 hours in the dark. Gallic acid was used as a reference standard. Some water soluble free polyphenols (pyrogallol, hydroxytyrosol, pyrocatechol and oleuropeine) were quantified by HPLC in the same solution.

HPLC analysis of monomer phenols were performed on a Varian 920 LC using a Varian Pursuit C18 column (125 × 4.6 mm, 5 μm) and a UV-DAD detector. The injection volume was 20 μl and elution was performed at a flow rate of 1.0 ml/min using 0.1% phoshoric acid (Merck) in water (solvent A) and 70% acetonitrile (Chemlab) in water (solvent B). The column temperature was maintained at 40°C. The gradient conditions were as follow: 0–25 min, 10-25% B; 25–35 min, 25-80% B; 35–37 min, 100% B and finally washing and reconditioning steps of the column (40–50 min, 100-10% B). The identification and quantification of phenolic compounds were based on their spectra, their retention times by comparison with phenolic standards (SIGMA) analyzed in the same conditions. The quantification wavelengths were: gallic acid (271 nm); hydroxytyrosol (280 nm); pyrocatechol (275 nm); pyrogallol (265 nm) and oleuropein (231 nm).

### Biogas and methane yields

The BMP assays of leaves (MU or MI) were determined following the procedure described by Rodriguez et al. (2005) and Wang et al. (1994). The tests were carried out in duplicate in 250 ml sterile glass serum bottles filled with 150 ml of a mixture. This mixture consisted of 125 ml of phosphate – carbonate buffer solution (with the pH adjusted to 7.2 with KOH 5 N), 25 ml of anaerobic sludge inoculum and milled leaves. Leaves grounded at 1 mm size were used for tests with 250 mg, 1000 mg, 2000 mg dry weight and shredded leaves at 2 cm size were used for the more concentrated test with 9200 mg raw matter. It is to note that the sludge was collected from a 20 liters stirred anaerobic digester used in Walloon Center of Industrial Biology for BMP assays of different agro-food organic wastes. This lab-scale digester was inoculated two years ago with a sludge collected from a full-scale anaerobic digester treating the activated sludge from a municipal waste water treatment plant. The minerals elements and vitamins were not added in the sample bottles considering that those substances should be present in the leaves. Each positive control sample consisted of 0.5 g of glucose monohydrate (Gl) alimented in two times (0.25 g at the beginning and 0.25 g after the 100th day, by adding 2 ml of a 125 g/l aqueous solution by syringe injection through the septum) and 25 ml of inoculum in a 250 ml sterile glass serum bottle containing 125 ml of phosphate – carbonate buffer solution. It is to note that 2 ml of the same Gl aqueous solution that the previous, was also added in the samples of MI leaves after the 100th day of the same manner that in positive control samples. This Gl addition was a test to discover the reasons of the methanogenesis inhibition. Each blank sample consisted of 25 ml of the anaerobic sludge inoculum and 125 ml of phosphate – carbonate buffer solution. No energetic substrate was added to the blank samples.

When the sample bottles were filled, they were capped tightly with rubber septa and sealed with aluminum seals, and nitrogen was passed into the bottles to flush out air and other gases before the incubation (Hiligsmann et al. 2011). The bottles were then incubated at 30°C, and the composition and volume of biogas produced were periodically measured during 230 days according to the method of CO_2_ absorption by KOH, described by Hiligsmann et al. (2011).

Biogas or methane yield was calculated by dividing the measured biogas or methane volume by the theoretical biogas or methane potential from the TOC content of each bottle. The maximum methane production rate was determined as the maximum slope from the cumulative methane production curve.

### Evolution of glucose, ethanol and VFAs

The evolution of glucose, ethanol and VFAs concentrations in samples was analyzed by HPLC. The samples were centrifuged at 13000 g for 10 min and the supernatants were filtered through a 0.2 μm cellulose acetate membrane (Sartorius Minisart). The glucose, ethanol, formate, acetate, propionate, butyrate, lactate and succinate were analyzed using a HPLC equipped with a differential refraction index detector as formerly described by Masset et al. (2010).

### Analysis of liquid and solid digestates

After 230 days of anaerobic digestion, the contents of BMP tests were separated in liquid and solid residues by centrifugation and filtration on 0.2 μm cellulose acetate membrane. The solid residues were dried for TOC and TKN analyses.

## Results

### Leaves characteristics

The results of physico-chemical analyses of leaves contents in dry weight, ash and organic matter, TOC, TKN and mineral elements are shown in Table 1. The C/N ratio was obtained from the ratio between the TOC and TKN contents and they were approximately of 7 and 48 for MU and MI leaves, respectively.

**Table 1:** **Physico-chemical characterization of leaves of MU and MI: dry weight content (DW), ashes and organic matter or volatile solid (VS), total organic carbon (TOC), total Kjeldahl nitrogen (TKN) and mineral elements**

**Components**	**Leaves**	
	MU	MI
Dry weight (%)	80.76 ± 0.04	88.68 ± 0.39
Organic matter (% DW)	85.17 ± 0.28	90.25 ± 0.05
Ashes (% DW)	14.83 ± 0.28	9.75 ± 0.05
TOC (mg/g DW)	357.49 ± 14.25	411.55 ± 12.48
TKN (mg/g DW)	50.50 ± 0.80	8.45 ± 0.29
P (mg/g DW)	2.23	0.01
K (mg/g DW)	21.49	6.29
Ca (mg/g DW)	13.89	27.79
Mg (mg/g DW)	4.51	0.92
Fe (mg/g DW)	0.41	0.08
Cu (mg/g DW)	0.02	<0.01
Mn (mg/g DW)	0.34	0.04
Zn (mg/g DW)	0.11	0.04

The results of qualitative identification of bioactive substances in aqueous extracts of leaves and further quantitative analysis of total polyphenols and some free specific polyphenols amounts are reported in Table 2. The saponins and 2 mg polyphenols (catechic tannins)/g were founded in the MU leaves. No free polyphenol was present in MU leaves among those were analyzed. The MI leaves contained saponins, anthraquinones and 20 mg polyphenols/g like flavonoids, anthocyanins, leuco-anthocyanins, gallic and catechic tannins. Among the analyzed free polyphenols only gallic acid, hydroxytyrosol, pyrogallol and pyrocatechol were present in MI leaves.

**Table 2:** **Bioactive substances and specific and total polyphenols (equivalent gallic acid per g of leaves) in leaves of MU and MI**

**Components**	**Leaves**	
	MU*	MI*
Saponins	++	+
Flavonoids	-	+
Alkaloids	-	-
Anthraquinones (bound quinones)	-	+
Catechic tannins	+	++
Gallic tannins	-	+
Anthocyanins	-	-
Leuco-anthocyanins	-	-
Total polyphenols (mg/g DW)	2.0	20.0
Gallic acids (mg/g DW)	0.0	5.8
Hydroxytyrosol (mg/g DW)	0.0	0.6
Pyrogallol (mg/g DW)	0.0	9.2
Pyroctechol (mg/g DW)	0.0	0.4
Oleuropeine (mg/g DW)	0.0	0.0

*- : absence; +: presence; ++: considerable presence.

### Evolution of the anaerobic digestion of MI and MU leaves

#### Biogas and methane yields

**Figure 1:**
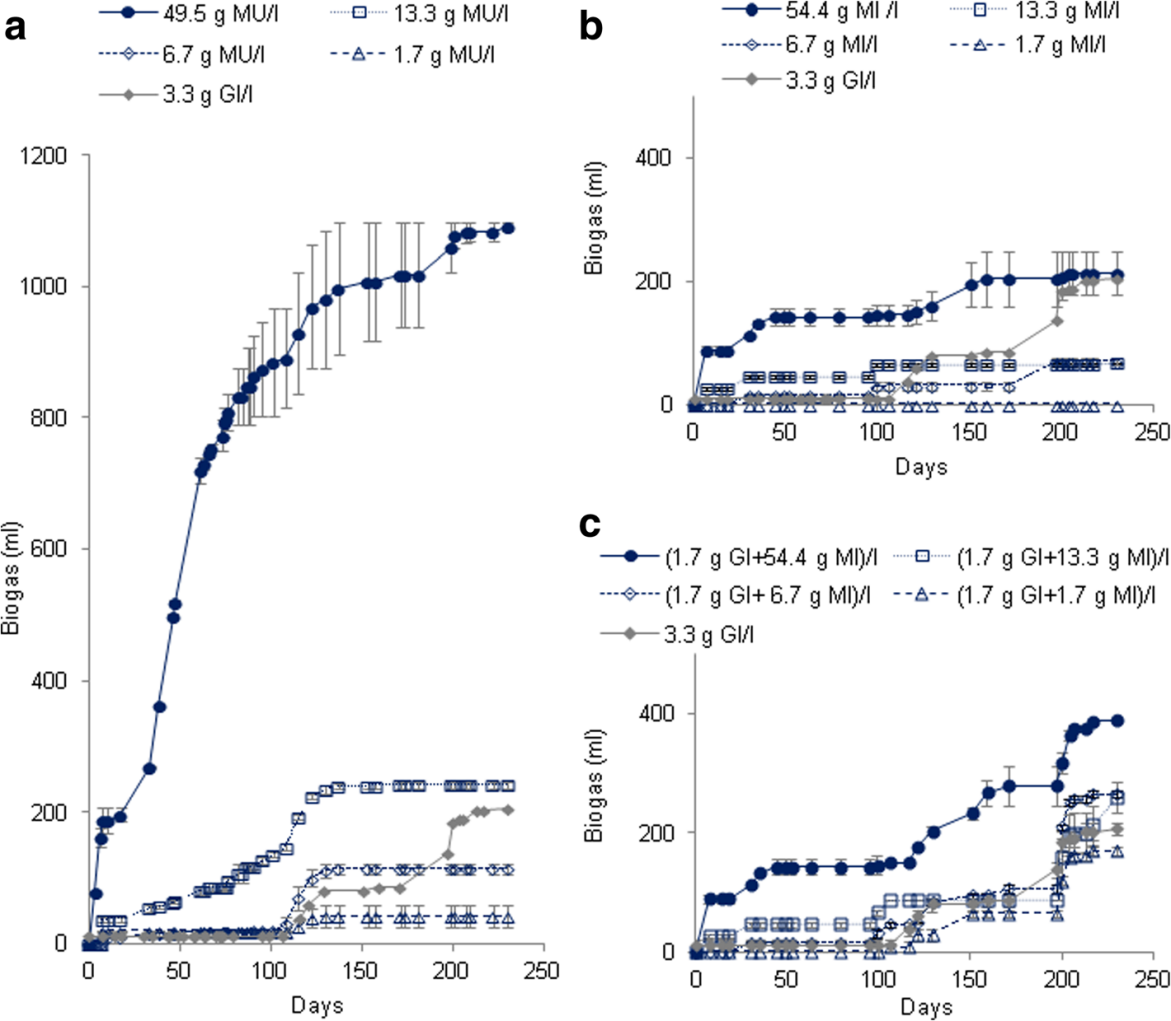
**Production of biogas (ml ± SD) during the anaerobic digestion of MU leaves alone (a), MI leaves alone (b) and MI leaves with glucose added after the 100th day to discover the reasons of the methanogenic inhibition observed (c) in BMP tests.**

The evolution of the biogas production was monitored in BMP tests carried out to assess anaerobic digestion of MU and MI leaves. The results are presented in Figure 1. No biogas was detected for blank samples. After 230 days of anaerobic digestion, the total volumes of biogas were 206 ± 10.5 ml for the Gl samples at concentration of 3.4 g/l, from 40.5 ± 17.5 to 1091.0 ± 5.8 ml for MU leaves at concentrations of 1.7 g/l and 49.5 g/l, respectively and from 0.0 ± 0.0 to 213.0 ± 35.0 ml for MI leaves at concentrations of 1.7 g/l and 54.4 g/l, respectively (Figure 1a,b).

The biogas production after the addition of Gl in the MI leaves samples after the 100th day were from 158.8 ± 6.8 to 243.3 ± 1.6 ml for the mixtures (MI + Gl) at concentrations of (1.7 g MI/l + 1.7 g Gl/l) and (54.4 g MI/l + 1.7 g Gl/l), respectively (Figure 1c).

**Figure 2:**
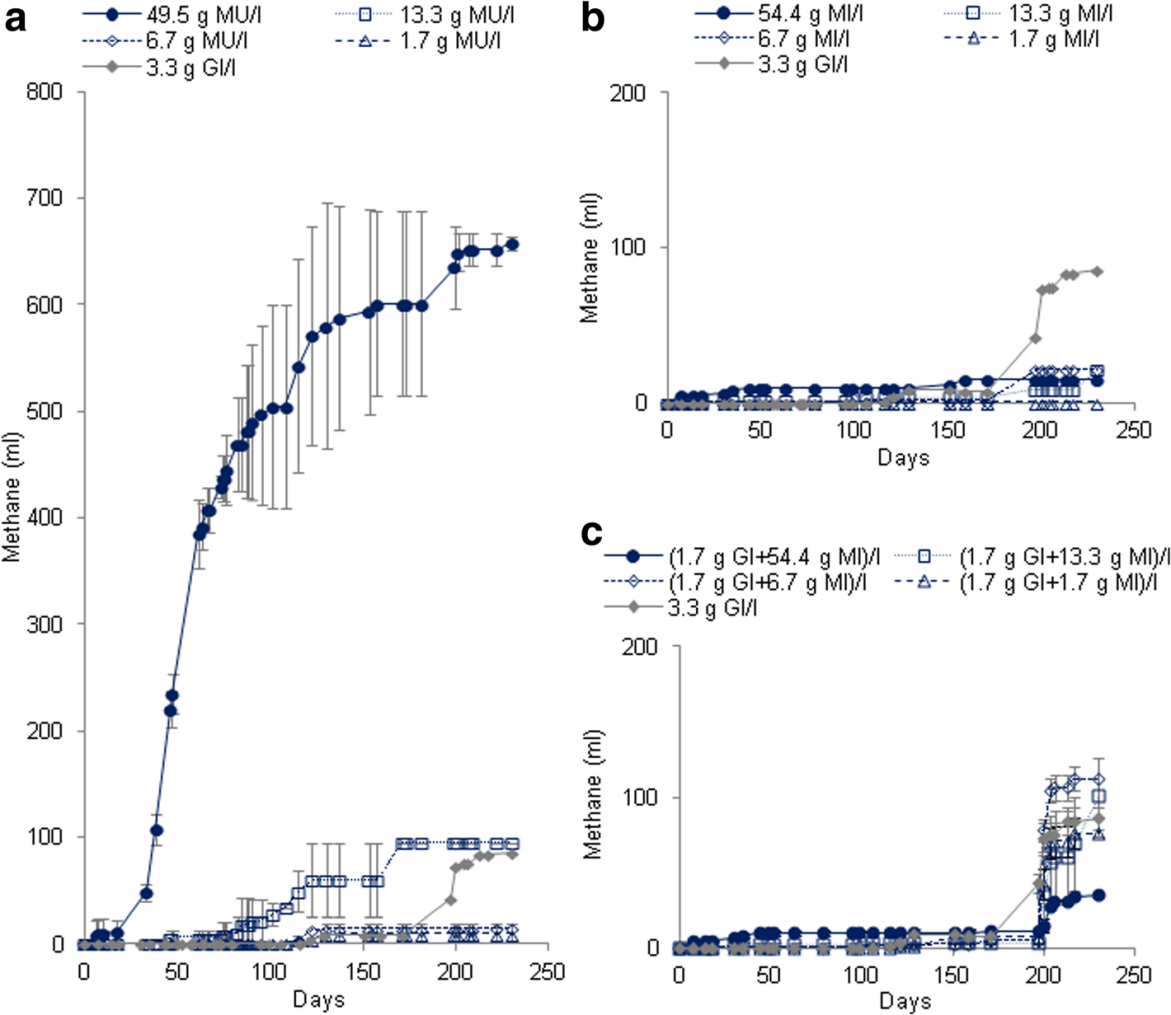
**Production of methane (ml ± SD) during the anaerobic digestion of MU leaves alone (a), MI leaves alone (b) and MI leaves with glucose added after the 100th day to discover the reasons of the methanogenic inhibition observed (c) in BMP tests.**

The results of methane production are showed in Figure 2. No methane was detected for blank samples. The total volumes methane were 85.8 ± 7.2 ml for Gl samples at concentration of 3.4 g/l, from 9 ± 10 to 658 ± 6 ml for MU leaves at concentrations 1.7 g/l and 49.5 g/l, respectively and from 0 ± 0 to 16 ± 7 ml for MI at concentrations of 1.7 g/l and 54.4 g/l, respectively (Figure 2a,b).

The methane production after the addition of Gl in the MI samples after the 100th day were from 75.9 ± 9.9 to 24.8 ± 1.6 ml for the mixtures (MI + Gl) at concentrations of (1.7 g MI/l + 1.7 g Gl/l) and (54.4 g MI/l + 1.7 g Gl/l), respectively (Figure 2c).

The volumetric biogas and methane production were expressed in terms of yields related to the initial TOC of the leaves and are reported in Table 3. The maximum methane production rates calculated for the different concentrations of MU leaves were: 0.39, 0.96, 1.00 and 12.15 ml/day at concentrations of 1.7, 6.7, 13.3 and 49.5 g/l, respectively. The energy amounts available in the biogas calculated from the calorific energy of methane produced from 1 kg of leaves are reported in Table 4.

**Table 3:** **Biogas and methane production yields after 230 days of BMP tests at 30°C with Gl, MU and MI leaves at concentrations of 1.7 g/l to 54.4 g DW/l**

**Samples concentrations**	**Biogas yields (ml/g TOC)**	**Methane yields (ml/g TOC)**
3.4 g Gl/l	159	68
1.7 g MU/l	453	101
6.7 g MU/l	297	92
13.3 g MU/l	338	131
49.5 g MU/l	411	248
1.7 g MI/l	0	0
6.7 g MI/l	167	50
13.3 g .MI/l	81	25
54.4 g MI/l	62	5

**Table 4:** **Energy amounts in the resulting biogas production from the anaerobic digestion of 1 kg of MU and MI leaves during 100 and 230 days**

**Samples concentrations**	**Methane yields for 100 days (l/g SV)**	**Methane yields for 230 days (l/g SV)**	**Energies for 100 days (kJ)**	**Energies for 230 days (kJ)**
1.7 g MU/l	0	0.042	0	1357.0
6.7 g MU/l	0	0.036	0	1163.1
13.3 g MU/l	0.023	0.055	743.1	1777.0
49.5 g MU/l	0.1	0.104	3230.9	3360.2
1.7 g MI/l	0	0	0	0
6.7 g MI/l	0	0.023	0	703.2
13.3 g MI/l	0.004	0.012	122.3	366.9
54.4 g MI/l	0.001	0.002	30.6	61.1

According to Shuku (2011), calorific power of methane is 37580 kJ/m^3^

#### Analysis of glucose, ethanol and volatile fatty acids (VFAs)

Concentrations of VFAs, glucose and ethanol in the culture media were measured by HPLC. The VFAs (succinic, formic, acetic, lactic, propionic and butyric acids), glucose and alcohol were released from leaves biodegradation. The glucose, ethanol, succinic and lactic acids were absent in the media.

**Figure 3:**
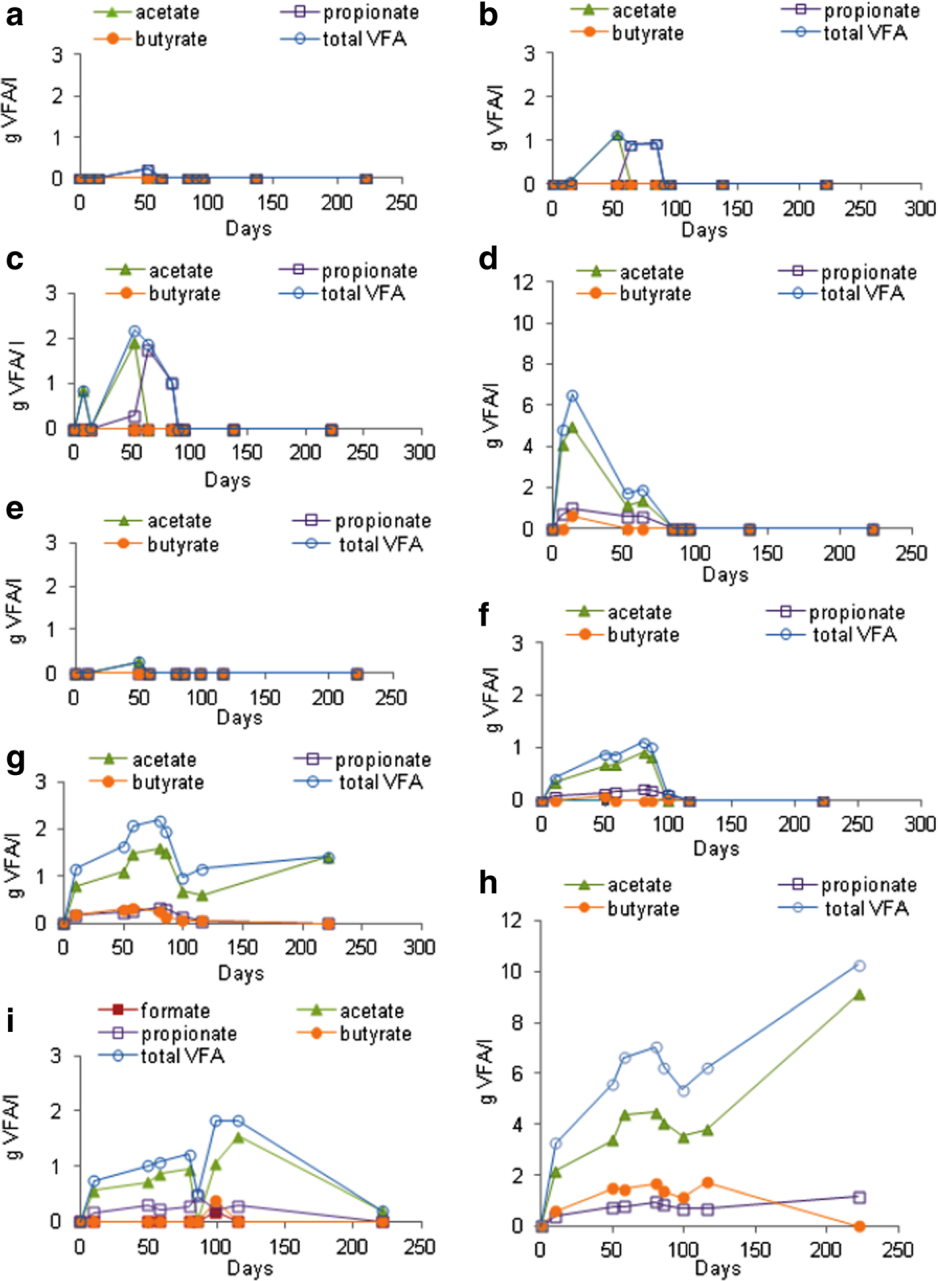
**VFAs production during anaerobic digestion of leaves in different concentrations in dry matter; MU leaves: a 1.7 g/l, b 6.7 g/l, c 13.3 g/l, d 49.5 g/l and MI leaves: e 1.7 g/l, f 6.7 g/l, g 13.3 g/l, h 54.4 g/l and i glucose in BMP tests.**

No VFA was detected in the blank samples. The maximum concentrations of VFAs reached to 1.5 g/l for test with Gl, from 0.2 to 6.5 g/l for MU leaves at concentrations of 1.7 g/l and 49.5 g/l, respectively and 0.3 to 10.3 g/l for MI leaves at concentrations of 1.7 g/l and 54.4 g/l, respectively (Figure 3). After 230 days, the concentrations of VFAs were 0 for all concentrations of MU leaves and both the lower concentration of MI leaves. By contrast, they were of 0.2 g/l for the positive test with Gl and of 1.4 and 10.3 g/l for MI leaves at concentrations of 13.3 g/l and 54.4 g/l, respectively (Figure 3). Acetic acid was the only one present VFA in Gl after 230 days.

**Figure 4:**
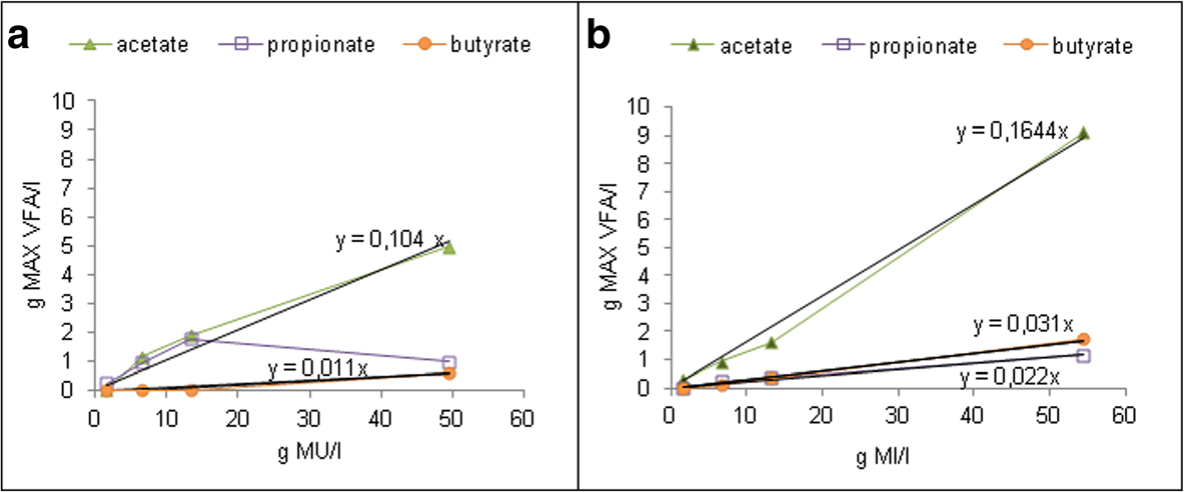
**Maximum concentration of each metabolite produced by anaerobic digestion from different concentrations (1.7 to 54.4 g DW/l) of MU leaves (a) and MI leaves (b) in BMP tests.**

The maximum amounts of the acetic acid were 1.5 g/l for Gl samples, from 0 to 5 g/l for MU leaves at concentrations of 1.7 g/l and 49.5 g/l, respectively and from 0.3 to 9.1 g/l for MI leaves at concentrations of 1.7 g/l and 54.4 g/l, respectively (Figure 4).

### Solid and liquid residues produced after the anaerobic digestion

After the anaerobic digestion, the residues were separated and their TOC and TKN were determined. C/N ratio of the residues were 1.58, 11.79, 63.64 and 48.11 for liquid residue of MU leaves, solid residue of MU leaves, liquid residue of MI leaves and solid residue of MI leaves, respectively (Table 5).

**Table 5:** **TOC and TKN contents in the solid and liquid residues produced after the anaerobic digestion in BMP test with 49.5 g MU/l and 54.4 g MI/l**

**Components**	**Digestates states**			
	Liquid MU*	**Solid MU**	Liquid MI*	**Solid MI**
TOC (mg/l*or mg/g)	1333.50 ± 11.50	428.50 ± 0.00	3354.00 ± 0.00	420.00 ± 0.00
TKN (mg/l*or mg/g)	843.00 ± 13.00	36.35 ± 0.35	52.70 ± 0.00	8.73 ± 0.38
C/N	1.58	11.79	63.64	48.11

*: Unit used for the components contained in the liquid digestates.

## Discussion

### Biogas yields

The total biogas volumes produced after 230 days of incubation increased with leaves concentrations (Figure 1). This trend was confirmed by the yields (Table 3) which were relatively similar from 310 to 411 ml/g TOC for MU leaves and from 0 to 62 ml/g TOC for MI leaves. They represented about 17 to 22% of the theoretical yields for MU leaves. That corresponded to the production of half a mole of methane and half a mole of carbon dioxide from one mole TOC. By contrast, the yields recorded for MI leaves represented about 9% of the theoretical yield at a concentration of 6.7 g leaves/l and not more than 3% at higher concentrations. A delay before start of biogas production should also be linked to leaves concentration. Indeed, the biogas production in BMP tests with 13.3 and 49.5 g MU/l produced biogas rapidly after inoculation whereas the experiments with lower DW contents began to produce biogas after more than 3 months of incubation although pH conditions were suitable, between 6.5 and 7.2. These results showed that leaves specific size of 2 cm in the test with 49.5 g/l for MU leaves and also 1 mm in the tests with lower concentrations had no effect on the biogas production and confirmed the hypothesis that particle sizes in the millimeter to centimeter range would not significantly expose more surface area and would thus exhibit similar kinetics (Chynoweth et a1. 1993). It should also be noticed that for MI leaves no biogas production was recorded at concentration of 1.7 g/l. Moreover, the cumulative biogas curve with 6.7 g MI/l overlapped that with 13.3 g/l after 197 days of digestion.

### Methane yields

Regarding cumulative methane production depicted in Figure 2, only a few CH_4_ could be produced from 1.7 g MU/l, 6.7 g MU/l and the experiments with MI leaves. Moreover, CH_4_ was only detected in biogas from the 6th and 46th day of culture for the MU leaves at concentrations of 49.5 and 13.3 g/l, respectively while biogas production has started from the beginning of incubation. These results suggest that about 1.7 g MU/l and 6.7 g MU/l are needed to enable sufficient growth and respiration (producing mainly CO_2_) of microflore before methane production if any from still available carbon. By contrast, the high methane production rate of 12.15 ml/day measured at concentration of 49.5 g MU/l suggests that a highly efficient anaerobic digestion occurred without limitations although the leaves were not fine shredded and their C/N ratio of about 7 (Table 1) was significantly different regarding the range from 20 to 30 commonly recommended for optimal anaerobic digestion (Mital 1996). Therefore, these results would suggest that a lack of nutrients or of suitable environmental conditions for methanogenesis was evidenced in the BMP tests with MU leaves at concentrations lower than 13.3 g DW/l or with MI leaves at any concentrations. The methane yields reported in Tables 3 and 4 confirmed this hypothesis since when comparing to the biogas yield of about 20%, related to theoretical biogas production, only the experiment with MU leaves at concentration of 49.5 g/l reaches a similar level of 27% CH_4_ yield related to theoretical methane production. Even the BMP test with MU leaves at 13.3 g/l achieved a quite low relative CH_4_ yield of 14%. By comparison to other leaves mentioned in the literature according to Gunaseelan (2004), only the yield at the concentration of 49.5 g MU/l was in the range but it was slightly low.

Although leaves of MI had similar organic matter content than leaves of MU (Table 1), they have produced a quite lower biogas volume for 230 days of anaerobic biodegradation. The extensive investigation of the leaves composition showed that the MI leaves contained lower amounts of nitrogen, mineral elements in general and microelements in particular and high presence of various bioactive components when comparing to MU leaves (Tables 1 and 2). Except for calcium that was half of the calcium content of MI leaves (Table 1). In addition to these substances identified in this work, MI leaves contain too the lignin (Nyamangara et al. 2009). Many papers had shown that lignin affects the digestibility and biogas production performance (Oliveira et al. 2007; Kamdem et al. 2013). Biogas production in Gl samples indicated that the inoculum contained nutrients capable to start the methanization. Indeed, the C/N ratio of 48 of MI leaves is high, comparatively to the ratios between 20 and 30 required for optimal anaerobic digestion (Mital 1996). However, these characteristics should not prevent methanogenic biodegradation to develop. This argument was confirmed by methane production from Gl and MI leaves at low concentrations for example at 6.7 g/l because the sludge (blank sample) would liberate ammonia that decreased the C/N ratio in these culture media in interaction with VFAs produced by MI leaves (Figure 2b,c). It is necessary to note that the inoculums-to-culture medium ratio was 1/6 (v/v). The low biogas or methane production from 6.7 g MI/l in relationship to its TOC content could be explained by the effects of saponins, anthraquinones and polyphenols (20 g/g). These substances contain hydrocarbon chain and cycle and, benzene rings that are difficult to be degraded by microorganisms; except, by specific microorganisms which seem not to be present in the inoculum or in the suitable conditions (e.g. aerobes). The methane yields for 100 days expressed according to Owen et al. (1979) and Gunaseelan (2004) for MI leaves are reported in Table 4. These yields would explain the low degradation kinetics or the low gas production (Hobson and Wheatley 1993) but not the total inhibition of the methanogenesis from MI leaves after a very few methane production (Figure 2b,c). After the 100th, Gl would also enable growth and metabolism of specific microorganisms able to further degrade MI leaves compounds or it would release nutrients enabling microorganisms to degrade MI leaves. In spite of it, the methane yields of MI leaves at different concentrations recorded were the lowest of those of known leaves. According to the review of Gunaseelan (2004), leaves with high yields of methane achieve about 0.430 l/g VS added and in general, the CH_4_ yield of leaves are in the range from 0.120 to 0.430 l/g VS. By comparison, Mahamat et al. (1989) reported methane yield of about 0.280 l/g VS for Calotropis, a plant from Sahel. They argued that this low yield would be due to the presence of some toxic compounds such as a strong cardiotonic that may partly inhibit the digestion process (Gunaseelan 1997).

By comparison to MI leaves, MU leaves produced biogas with a high energy amount at high concentrations representing up 7% of the calorific power of wood after 100 days (Table 4). By contrast, MI leaves produced a biogas with a weak energy amount at low concentrations nearly 2% of the calorific power of wood after 230 days (Table 4). It should be known that according to Shuku (2011), calorific powers of methane and wood are 37580 kJ/m^3^ and 16736 kJ/kg, respectively.

### Evolutions of glucose, ethanol and volatile fatty acids (VFAs)

In general, the total quantities of VFAs increased with the leaves amounts in the bottles. The concentrations were similar in the BMP tests carried out with the same leaves contents of MU or MI leaves. This suggests that hydrolysis and acidogenesis processes were efficient whatever the organic matter. This is confirmed by Figure 4 since, except for propionate produced by MU leaves, the maximum concentration of each VFA measured in the different BMP tests, was proportional to initial substrate concentration and similar trends were recorded for MU and MI leaves. The concentrations of MU or MI leaves (1.7 and 6.7 g/l) further were converted to biogas. Therefore, these results show that the low yields and conversion rates of MI leaves to methane would especially be due to the concentrations and the synergism of their bioactive compounds (Chen et al. 2008) and probably to carbon conversion for all biomass formation.

A few negative effects appeared in the MU leaves at low concentrations for instance at 6.7 g/l where the VFAs maximum production was low (Figures 3a,b and 4a). It was observed an increase of the pH from 7.4 to 7.9 that could be explained by the release of the ammonia in the culture medium thus also leading to a slow methane production (Figure 2a) and a low methane yield. The effect of free ammonia would become 0 at 13.3 and 49.5 g MU/l. It is necessary to know that the instability process due to ammonia often results in VFAs accumulation, which again leads to a decrease in pH and thereby declining concentration of free ammonia. Wherefore the interaction between free ammonia, VFAs and pH may lead to an “inhibited steady state”, a condition where the process is running stably but with lower methane yield (Chen et al. 2008; Angelidaki and Ahring 1993; Angelidaki et al. 1993). Furthermore, the propionate profile for MU leaves with a lower maximum production at 49.5 g/l than at 13.3 g/l (Figure 4) suggests the presence of improving substances for propionate conversion to acetate. Thus, all volatile fatty acids produced from MU biomass were converted to biogas even at the very high leaves concentration of 49,5 g/l although, by comparison to MI leaves, the leaves of MU contained a considerable amount of saponins and a total polyphenols content of 2 mg/g. According to Multon (1991), the saponins are minor compounds of plants.

By contrast, acetate accumulation was observed in the bottles containing MI leaves at 13.3 g/l and 54.4 g/l (Figure 3g,h). After 90 days of incubation, in the media of MI leaves at 13.3 g/l and 54.4 g/l, some amounts of acetate were consumed without significant production of neither CH_4_ and nor CO_2_. That could be related to the metabolic pathway of reversible homoacetogenic bacteria that are frequently detected in anaerobic digesters however their activity is not yet well understood (Luo et al. 2011; Wang et al. 2013). Accumulation of propionate in the bottles of MI leaves at 54.4 g/l was also recorded. Thus, there was not acetogenesis, nor methanogenesis obvious for MI leaves at 54.4 and g/l 13.3 g/l, respectively (Figure 3 g,h). That was observed from propionate accumulation. According to the literature, the accumulation of VFAs is an indicator of an inhibition (Chen et al. 2008). In our case, the accumulation of acetate and propionate would not be due to the high C/N ratio of MI leaves in these concentrations. The results obtained after the addition of glucose in the culture media after 100 days of incubation showed a further production of methane and biogas (Figures 1c and 2c). They demonstrate that these inhibitions were due not only to the high C/N ratio but especially to the increase of contents in bioactive matters of MI leaves in the culture media and to their synergic effect. Indeed, the higher concentration of 10.3 g VFAs/l observed in Figure 3h, would not completely inhibit the methanization according to Buffiere et al. (2007). Furthermore, these inhibitions were partial. That could be explained by the presence of some resistant methanogenic bacteria in culture media such as methanobacterium formicium and methanococcus vannelli which transform H_2_, CO_2_ and formate into methane.

Consequently, only a little amount of the VFA produced from MI leaves were converted in methane comparatively to MU leaves. That might be correlated to the high content of MI leaves in various bioactive substances (saponins, anthraqunones, flavonoids, anthocyanins, leuco-anthocyanins, gallic and catechic tannins) and especially in 20 mg total polyphenols/g DW. These bioactive substances are inhibitory of methanogenesis (Macheboeuf et al. 2011; Patra and Saxena 2010; Kamra et al. 2008). Furthermore among the water-soluble polyphenols, MI contained a high quantity of pyrogallol (Table 2). This kind of monomeric phenols would be more inhibitive than the polymers (Hobson and Wheatley 1993). The aqueous extracts of MI leaves were already reported to be rich in polyphenols and to possess an antimicrobial activity (Masibo and He 2009; Nunez-Selles 2005). Indeed, aromatic ring compounds, particulary polyphenols may exert toxicity at 700 mg/l (Gerardi 2003). However, in this study, the tests of MI leaves at concentrations of 13.3 g/l and 54.4 g/l have given 267 mg/l and 1093 mg/l of polyphenols, respectively without taking into account other aromatic ring compounds such as the anthraquinones.

### Digestates

At the end of anaerobic digestion, the nitrogen concentration in the residual liquid solution of MU leaves was of 856 mg/l (Table 5); this concentration was lower than the inhibitory (1500 mg/l) at pH 6.5-7.2 reported by Gerardi (2003). That could explain why although the nitrogen content in MU leaves was high, there was no adverse effect (Gerardi 2003). The liquid residue of MU leaves with a C/N ratio of 1.58 was rich in nitrogen and so it could be used as fertilizer for plants (Hobson and Wheatley 1993). The solid residue of MU leaves had a C/N ratio of 11.79 that was similar to 10, considered as optimal for soil organisms and soil-conditioning (MCDF 1993; Ducat and Bock 1995; Davet 1996; Mze 2008). This C/N ratio would avoid competition between microorganisms and plants for their growths although the C/N ratio is not the index of the absolute quality of organic matter (Mze 2008). In general, digested sludges of plant are generally decomposed in the soil slower than are the original materials. This slow decomposition has advantage as it preserves the fibers structure as soil conditioner and leaves readily available ammonia nitrogen to plants instead of its being used by microbes growing rapidly on sludge constituents (Hobson and Wheatley 1993). Furthermore, these residues should contain mineral elements e.g. K (Table 1) and microorganisms able to boost the enzymatic and microbial activities in the soil. They would be good fertilizers for vegetables. They could be used without be separated by spreading them on the soil. By contrast, the solid residue of MI leaves with a C/N ratio of 48.11 and its liquid residue rich in carbon with a C/N ratio of 63.64 due to high content of VFAs (Figures 3 and 4) and bioactive substances. These residues cannot be used as fertilizer since they would cause pollution (Anid 1983; Hobson and Wheatley 1993). By contrast, the residues of MI leaves at concentration of 6.7 g/l, exempt of VFAs were a poor and inadequate source of nitrogen for plant growth in the short term. They could contribute to soil organic matter build-up in the long-term. According to the organic resource data base developed by Palm et al. (2001), these materials should be mixed with nitrogen fertilizer before application to soil in order to reduce the negative effects of nitrogen immobilization (Nyamangara et al. 2009).

## Conclusion

In this paper, BMP tests were carried out at mesophilic temperature (30°C) with leaves from two common tropical trees. The results proved that the methanogenic biodegradation of MU leaves alone is feasible for any amount of leaves up to 61.3 g/l shredded at 2 cm size or lower. Moreover, the methanogenic kinetic was very efficient at this amount and without addition of nutrients. However, the methane yield is relatively low, 0.1 l CH_4_/g VS for 100 days of incubation when comparing to other leaves. It could probably be due to the low C/N value and inability of microorganisms to degrade the cycles or chain from complex molecules such as saponins and polyphenols. However, theses bioactive organic molecules did not inhibit the anaerobic digestion process. Indeed, the residues from anaerobic digestion of MU leaves could represent efficient fertilizers for the promotion of the biological vegetable garden. Therefore, this study showed that it is possible to transform at the ambient temperature this waste biomass into biofuel and biofertilizer on the same time particularly in Kinshasa and generally in tropical regions. This could be beneficial in the context of eliminating the environmental pollution caused by this biomass. By contrast, whereas acidogenic biodegradation of MI leaves were observed at the concentrations of 13.3 g/l and 54.4 g/l including homoacetogenic metabolism. The methanogenesis seemed to be inhibited. Our results showed that this inhibition did not depend on the high C/N ratio nor on the generally low content in mineral elements but especially on the content of bioactive compounds such as saponins, anthraquinones and polyphenols (flavonoids, anthocyanins, leuco-anthocyanins, gallic and catechic tannins and other monomeric phenols indentified) and their synergic effect. At concentrations equal or higher than 13.3 g MI/l, the anaerobic digestion of MI leaves could be thus served to valorize the residual fluxes (volatile fatty acids and the aromatic compounds) of bio-cascading pathways. However, this work demonstrated the possibility to produce methane at concentrations less than 13.3 g/l of MI leaves without inhibition although with a low methane yield for example 0.023 l/g VS after 230 days. In this case, their residues were exempt of VFAs, poor in nitrogen and they would be better than compost of MI leaves and could contribute to soil build up organic matter or use in combination with other fertilizer.

Further investigations will be carried out on the improvement of anaerobic degradation of MI leaves at concentrations higher than 13.3 g/l and on the specific effects of bioactive substances on methanogenic biodegradation in order to determine the real inhibitors of anaerobic digestion of MI leaves and the way to overlap it to optimize the biodegradation of both leaves in co-digestion.

## Abbreviations

BMP: Biochemical methane potential
C/N: Carbon/nitrogen ratio
DAD: Diode array detector
DW: Dry weight
Gl: Glucose
HPLC: High performance liquid chromatography
MI: *Mangifera Indica*
MU: *Manihot Utilissima*
TKN: Total Kjeldahl nitrogen
SD: Standard deviation
UV: Ultraviolet
TOC: Total organic carbon
VFA: Volatile fatty acid
VS: Volatile solid
